# Fully automated calcium scoring predicts all-cause mortality at 12 years in the MILD lung cancer screening trial

**DOI:** 10.1371/journal.pone.0285593

**Published:** 2023-05-16

**Authors:** Federica Sabia, Maurizio Balbi, Roberta E. Ledda, Gianluca Milanese, Margherita Ruggirello, Camilla Valsecchi, Alfonso Marchianò, Nicola Sverzellati, Ugo Pastorino

**Affiliations:** 1 Department of Thoracic Surgery, Fondazione IRCCS Istituto Nazionale dei Tumori, Milan, Italy; 2 Section of Radiology, Department of Medicine and Surgery (DiMeC), University Hospital of Parma, Parma, Italy; 3 Department of Radiology, Fondazione IRCCS Istituto Nazionale dei Tumori, Milan, Italy; Sant Anna Hospital: Clinica Sant’Anna, SWITZERLAND

## Abstract

Coronary artery calcium (CAC) is a known risk factor for cardiovascular (CV) events and mortality but is not yet routinely evaluated in low-dose computed tomography (LDCT)-based lung cancer screening (LCS). The present analysis explored the capacity of a fully automated CAC scoring to predict 12-year mortality in the Multicentric Italian Lung Detection (MILD) LCS trial. The study included 2239 volunteers of the MILD trial who underwent a baseline LDCT from September 2005 to January 2011, with a median follow-up of 190 months. The CAC score was measured by a commercially available fully automated artificial intelligence (AI) software and stratified into five strata: 0, 1–10, 11–100, 101–400, and > 400. Twelve-year all-cause mortality was 8.5% (191/2239) overall, 3.2% with CAC = 0, 4.9% with CAC = 1–10, 8.0% with CAC = 11–100, 11.5% with CAC = 101–400, and 17% with CAC > 400. In Cox proportional hazards regression analysis, CAC > 400 was associated with a higher 12-year all-cause mortality both in a univariate model (hazard ratio, HR, 5.75 [95% confidence interval, CI, 2.08–15.92] compared to CAC = 0) and after adjustment for baseline confounders (HR, 3.80 [95%CI, 1.35–10.74] compared to CAC = 0). All-cause mortality significantly increased with increasing CAC (7% in CAC ≤ 400 vs. 17% in CAC > 400, Log-Rank p-value <0.001). Non-cancer at 12 years mortality was 3% (67/2239) overall, 0.8% with CAC = 0, 1.0% with CAC = 1–10, 2.9% with CAC = 11–100, 3.6% with CAC = 101–400, and 8.2% with CAC > 400 (Grey’s test p < 0.001). In Fine and Gray’s competing risk model, CAC > 400 predicted 12-year non-cancer mortality in a univariate model (sub-distribution hazard ratio, SHR, 10.62 [95% confidence interval, CI, 1.43–78.98] compared to CAC = 0), but the association was no longer significant after adjustment for baseline confounders. In conclusion, fully automated CAC scoring was effective in predicting all-cause mortality at 12 years in a LCS setting.

## Introduction

Coronary artery calcium (CAC) is an independent predictor of cardiovascular (CV) events and mortality [1]. Previous studies in low-dose computed tomography (LDCT) lung cancer screening (LCS) participants demonstrated the predictive value of manual CAC scoring [2, 3]. Nevertheless, the timely consuming procedure of manually computing CAC scoring currently hinders its routine evaluation in LCS. Artificial intelligence (AI) software has shown promise to provide fully automated CAC quantification, possibly refining post-test risk assessment in LCS while speeding up the process and minimizing variability [4]. However, the evidence in favor of such an approach is still limited, particularly in non-electrocardiogram (ECG)-gated LDCT.

The present study aimed at assessing the predictive value of an automated CAC quantification for 12-year all-cause and non-cancer mortality in the Multicentric Italian Lung Detection (MILD) LCS screening trial. The association between CAC and lung cancer mortality was also explored.

## Material and methods

### Study participants

Details about the MILD study (ClinicalTrials.gov Identifier: NCT02837809) were published elsewhere [5, 6]. Briefly, MILD eligibility criteria were as follows: age 49–75 years, current or former smokers (having quit smoking within 10 years before recruitment) with at least 20 pack-years of smoking history, and no history of cancer within the previous 5 years. The MILD project was initially designed as a multicentric trial, with a planned sample size of 10 000 individuals, a screening period of 10 years, and a total follow-up of 100 000 person-years. Such a sample size would be adequate to detect a 30% reduction in lung cancer mortality in the LDCT arm. However, the national program faced many difficulties as a result of a lack of funding, limited support from local authorities, and cultural prejudice: only a few hospitals from the Lombardy region obtained permission to start the trial, and recruitment was limited. For these reasons, we included in the MILD trial only the individuals enrolled and screened at the Istituto Nazionale Tumori of Milan. A total of 4099 participants were enrolled from September 2005 to January 2011. Of them, 1723 were randomized to the control group and 2376 to the LDCT group. All the MILD trial volunteers randomized to the LDCT group were considered potentially eligible in our retrospective study. The original Institutional Review Board approval and written informed consent allowed the use of data for future research, including the present analyses.

### Imaging acquisition and analysis

LDCTs were acquired on a 16–detector row CT scanner (Somatom Sensation 16; Siemens Medical Solutions, Forchheim, Germany). The whole chest volume was scanned during one deep inspiratory breath-hold without the use of a contrast medium and with the following scanning parameters: tube voltage, 120 kV; effective tube current, 30 mAs; individual detector collimation, 0.75 mm; gantry rotation time, 0.5 seconds; and pitch, 1.5. Neither ECG-triggering nor dose-modulation systems were used. Images were reconstructed as follows: one-millimeter-thick sections were reconstructed with an increment of 1 mm (medium-sharp kernel, B50f), and 5-mm-thick sections were reconstructed with an increment of 5 mm (medium-smooth kernel, B30f).

**Fig 1:**
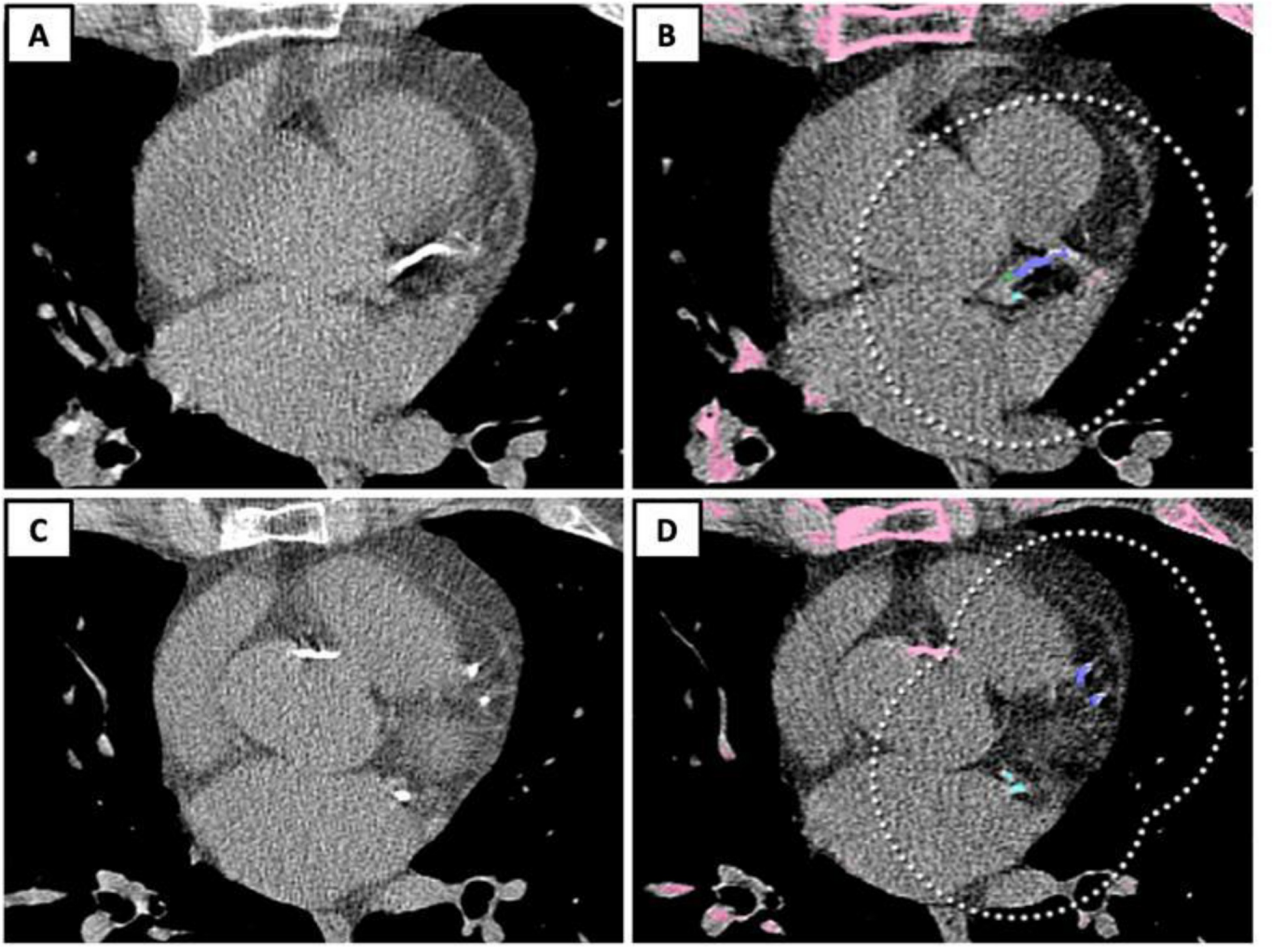
Magnification of LDCT images in the axial view (at two different anatomical levels) showing calcified left main coronary artery (A) and calcified left anterior descending and circumflex coronary arteries (C). In B and D the automated segmentation performed by the software: left main and left anterior descending coronary arteries colored in *purple* in B and D respectively, and circumflex coronary artery colored in *green* (D) for a total Agatston score of 460. It should be noted that the aortic valve calcifications are not segmented by the software.

For the automated CAC evaluation, 1-mm images were transferred to a dedicated graphic station (Alienware Area 51 R6 equipped with Dual NVIDIA GeForce RTX 2080 OC graphics) and analyzed using commercial AI software (AVIEW, Coreline Soft, Seoul, Korea) based on a 3-dimensional U-net architecture [7] (Fig 1). The rationale for using 1-mm images was supported by previous data demonstrating a more accurate CAC scoring with 1-mm than thicker slices in LCS LDCTs [8, 9]. CAC was assessed using the Agatston score and stratified into the following strata: 0, 1–10, 11–100, 101–400, and > 400 [10].

### Demographic, clinical, and follow-up data

Clinical and demographic information was retrieved by a medical doctor from the written questionnaire completed by each study participant during the baseline visit. The vital status and date of death were obtained through the Istituto Nazionale di Statistica (ISTAT, SIATEL 2.0 platform). Participants accumulated person-years of follow-up from the date of baseline until death or the date of the last follow-up as of August 2022. Causes of death were collected through direct contact with referring mortality and cancer registers located throughout Italy. A total of 17/191 causes of death were missing.

### Statistical analysis

Categorical variables were reported as numbers and percentages, whereas continuous variables as medians with interquartile ranges (IQRs); associations were evaluated by the Cochran-Mantel- Haenszel Test for trend for categorical data and by the Jonckheere-Terpstra Test for trend for continuous variables. Boxplots were reported to describe the distribution of CAC scores stratified by (i) age (<55, 55–59, 60–64, and ≥ 65) and sex and by (ii) pack-years (<30, 30–39, 40–49, and ≥ 50) and sex. Kaplan-Meier curve for 12-year all-cause mortality was reported in strata of CAC score in all participants, and comparisons were tested by Log-Rank test for trend. Twelve-year non-cancer mortality was estimated by cumulative incidence function for competing risk, and comparisons were tested by Gray’s test. Mortality curves were also stratified by sex. Lung cancer-specific mortality was explored as a supplementary analysis by cumulative incidence function for competing risk, and comparisons were tested by Gray’s test. Univariate and multivariate Cox proportional hazard regression was applied to estimate the 12-year all-cause mortality hazard ratio (HR) and 95% confidence interval (CI). Univariate and multivariate Fine and Gray’s competing risk model was used to estimate the 12-year non-cancer mortality sub-distribution hazard ratio (SHR) and 95% confidence interval (CI). Multivariate models were adjusted for age, sex, smoking status, pack-years, body mass index (BMI), and prior CV disease (i.e., angina, myocardial infarction, stroke, or thrombosis) to reduce the potential effect of different baseline characteristics. The analyses were performed using the Statistical Analysis System Software (Release SAS:9.04; SAS Institute, Cary, North Carolina, USA) and R Statistical Software (R Studio).

## Results

One hundred thirty-seven (5.8%) subjects were excluded due to incomplete data (86/2376) or software failure (51/2376). The final study population comprised 2239 participants: 62% were < 60 years old, 68% were males, and 68% were current smokers (Table 1). The median and minimum follow-up time for alive volunteers were 15.9 years (IQR, 15.7–16.3) and 11.8 years, respectively.

**Table 1:** Characteristics of the study population according to the AI-based automated CAC scores.

		Total		CAC score				Trend test p-value
			0	1–10	11–100	101–400	>400	
		2239	125 (5.6%)	698 (31.2%)	730 (32.6%)	393 (17.6%)	293 (13.1%)	
**Age**	**< 60**	1392 (62.2%)	93 (6.7%)	512 (36.8%)	479 (34.4%)	186 (13.4%)	122 (8.8%)	**<0.001**
	**≥ 60**	847 (37.8%)	32 (3.8%)	186 (22.0%)	251 (29.6%)	207 (24.4%)	171 (20.2%)	
**Sex**	**Male**	1524 (68.1%)	57 (3.7%)	363 (23.8%)	520 (34.1%)	324 (21.3%)	260 (17.1%)	**<0.001**
** **	**Female**	715 (31.9%)	68 (9.5%)	335 (46.9%)	210 (29.4%)	69 (9.7%)	33 (4.6%)	
**Smoking Status**	**Ex Smoker**	710 (31.7%)	37 (5.2%)	183 (25.8%)	233 (32.8%)	139 (19.6%)	118 (16.6%)	**<0.001**
** **	**Current Smoker**	1529 (68.3%)	88 (5.8%)	515 (33.7%)	497 (32.5%)	254 (16.6%)	175 (11.5%)	
**Pack-years**	**<30**	497 (22.2%)	36 (7.2%)	169 (34.0%)	154 (31.0%)	87 (17.5%)	51 (10.3%)	**0.0070**
	**≥30**	1742 (77.8%)	89 (5.1%)	529 (30.4%)	576 (33.1%)	306 (17.6%)	242 (13.9%)	
**BMI**	**Median (IQR)**	25.7 (23.4–28.4)	23.2 (20.7–25.7)	24.7 (22.7–27.3)	26.4 (24.1–28.7)	25.9 (24.1–29.0)	26.8 (24.5–29.1)	**<0.001**
**CVD** ^**a**^		217 (9.7%)	10 (4.6%)	49 (22.6%)	60 (27.7%)	40 (18.4%)	58 (26.7%)	**<0.001**
**All-cause mortality** ^**b**^		191 (8.5%)	4 (2.1%)	34 (17.8%)	58 (30.4%)	45 (23.6%)	50 (26.2%)	**<0.001**
**Non-cancer mortality**		67 (3.0%)	1 (1.5%)	7 (10.5%)	21 (31.3%)	14 (20.9%)	24 (35.8%)	**<0.001**
**Lung cancer monrtality**		51 (2.3%)	1 (2.0%)	12 (23.5%)	18 (35.3%)	9 (17.7%)	11 (21.6%)	**0.0377**

IQR, Interquartile Range; BMI, Body Mass Index; CVD, Cardiovascular Disease

^a^Myocardial infarction, stroke, thrombosis, or angina

^b^ 17 missing causes of death.

**Fig 2:**
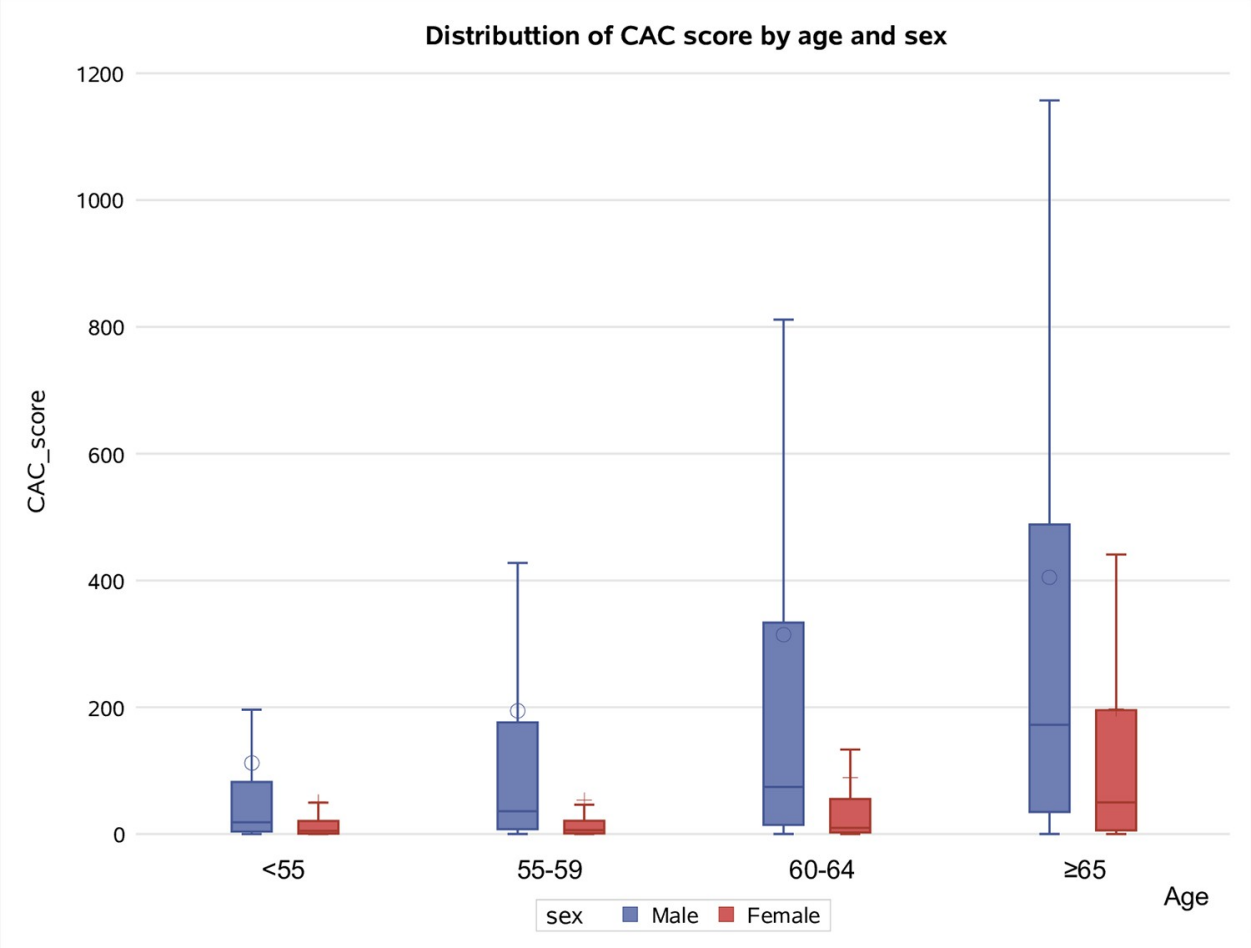
Distribution of CAC score by age and sex.

**Fig 3:**
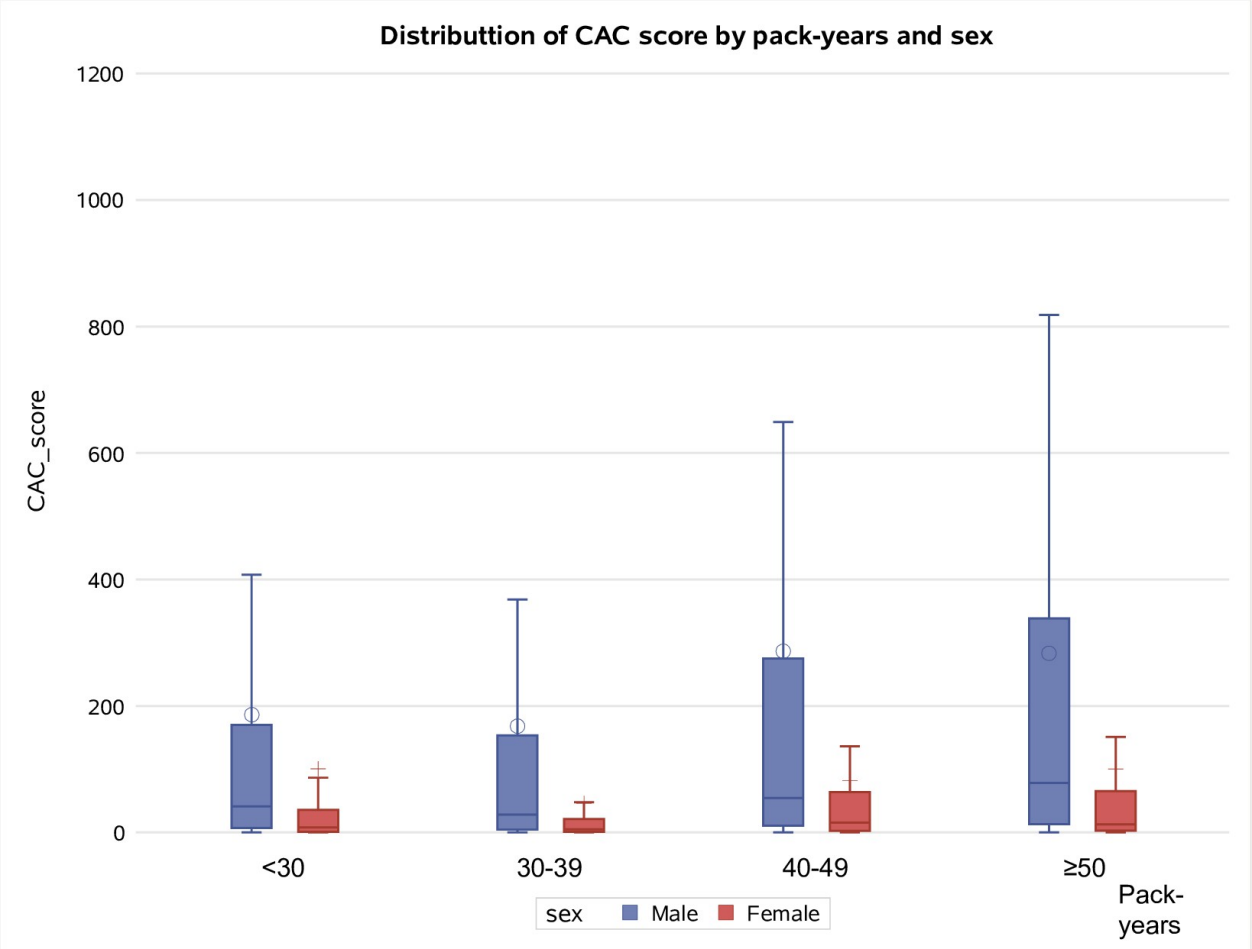
Distribution of CAC score by pack-years and sex.

CAC score was 0, 1–10, 11–100, 101–400, and > 400 in 125 (5.6%), 698 (31.2%), 730 (32.6%), 393 (17.6%), and 293 (13.1%) cases, respectively. CAC score was significantly higher in males than in females (p < 0.001) and in participants aged ≥ 60 (p < 0.001). Significant differences were also observed for smoking status (17% of ex-smokers had CAC > 400 compared to 11% of current smokers, p < 0.001) and pack-years (participants with ≥ 30 pack-years had a higher frequency of high CAC scores, p = 0.007). The median BMI increased as the CAC score increased (p < 0.001), as well as the presence of prior CV diseases (p < 0.001) (Table 1). CAC score was higher in males than in females and increased as the age and pack-years increased in both sexes (Figs 2 and 3).

Twelve-year all-cause mortality was 8.5% (191/2239) overall, 3.2% (4/125) with CAC = 0, 4.9% (34/698) with CAC = 1–10, 8.0% (58/730) with CAC = 11–100, 11.5% (45/393) with CAC = 101–400, and 17% (50/293) with CAC > 400. Twelve-year non-cancer mortality was 3% (67/2239), 0.8% (1/125) with CAC = 0, 1.0% (7/698) with CAC = 1–10, 2.9% (21/730) with CAC = 11–100, 3.6% (14/393) with CAC = 101–400, and 8.2% (24/293) with CAC > 400. Lung cancer-specific mortality was 2.3% (51/2239) overall, 0.8% (1/125) with CAC = 0, 1.7% (12/698) with CAC = 1–10, 2.5% (18/730) with CAC = 11–100, 2.3% (9/393) with CAC = 101–400, and 3.8% (11/293) with CAC > 400.

**Fig 4:**
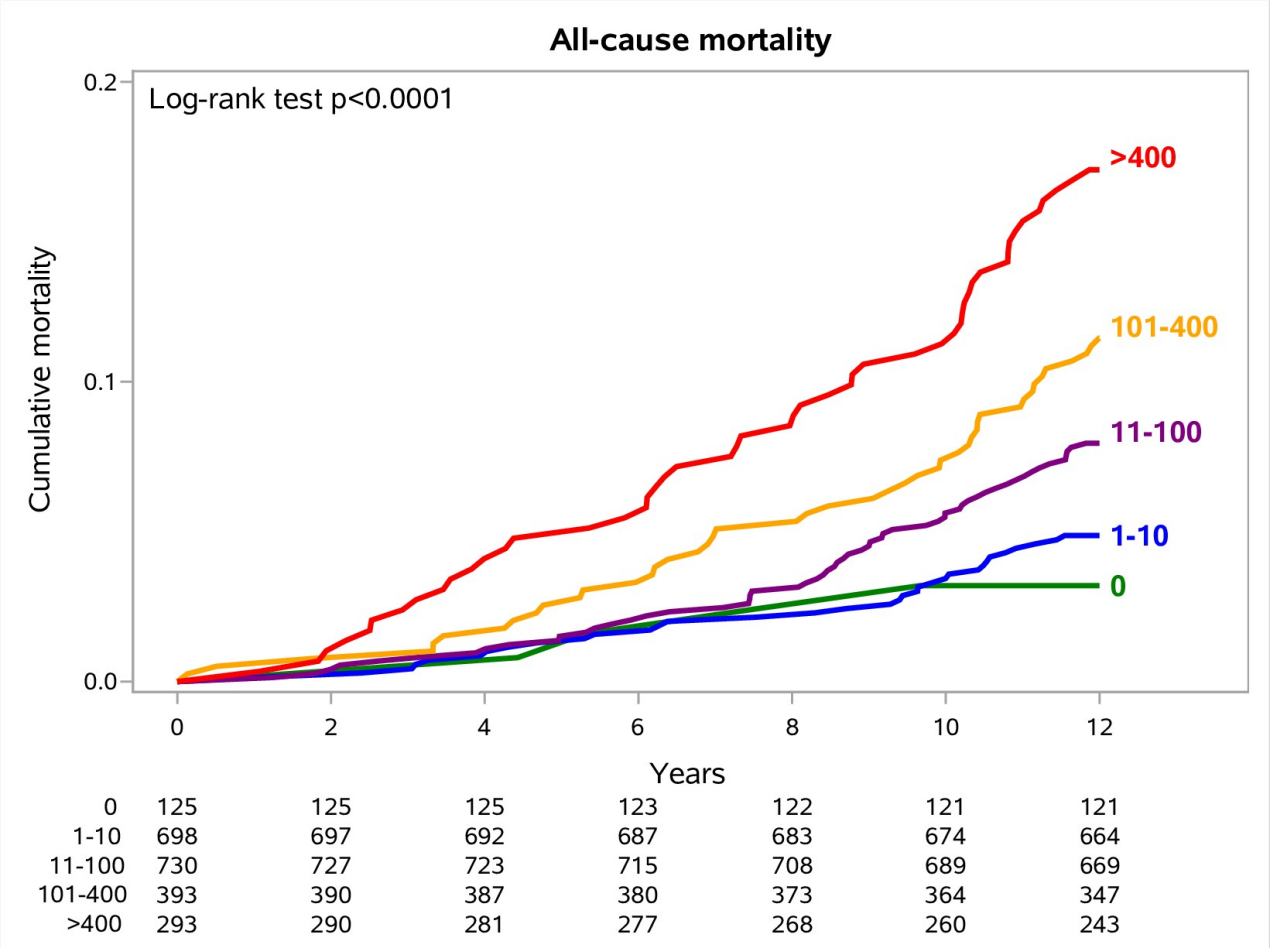
12-year all-cause mortality curves stratified by automated CAC scores.

**Fig 5:**
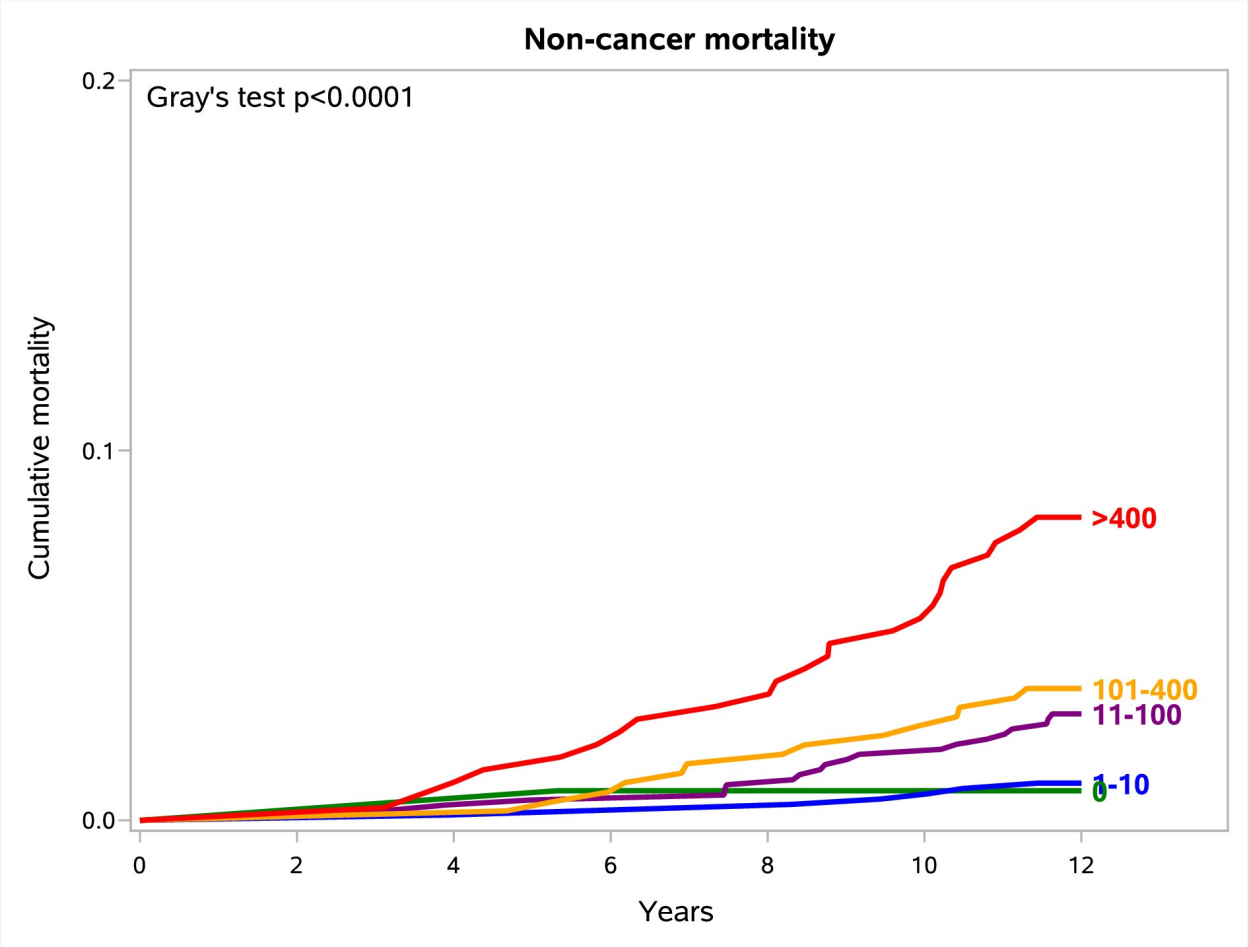
Cumulative incidence function of 12-year non-cancer mortality curves stratified by automated CAC scores.

In the 12-year all-cause mortality Cox proportional hazards regression model, unadjusted HRs were statistically significant for CAC 101–400 (HR, 3.71 [95%CI, 1.34–10.32]) and > 400 (HR, 5.75 [95%CI, 2.08–15.92]) (Table 2). In the multivariate model, a CAC score > 400 was still significantly associated with all-cause mortality (HR, 3.80 [95%CI, 1.35–10.74]). Among the baseline potential confounders, only an age ≥ 60 and being current smokers significantly predicted all-cause mortality (S1 Table in S1 File). The life expectancy at 12 years significantly decreased with higher CAC (Log-Rank test, p < 0.001) (Fig 4). The same trend was confirmed by analyzing males and females separately (S1A and S1B Fig in S1 File, respectively). Volunteers with a CAC ≤ 100 had a lower risk of all-cause mortality than subjects with a CAC > 100 (6% vs. 14%, Log-Rank p < 0.001), as well as those with a CAC ≤ 400 as compared with those with a CAC > 400 (7% vs. 17%, Log-Rank p < 0.001). The risk of non-cancer mortality significantly increased with increasing CAC (Gray’s test, p < 0.001) (Fig 5). In 12-year non-cancer Fine and Gray’s competing risk model, unadjusted SHR was statistically significant for CAC > 400 (SHR, 10.62 [95%CI, 1.43–78.98]) (Table 3). In the multivariate model, the CAC score > 400 was no longer statistically significant. The increased risk was shown both in males and in females separately, despite the decrease in the number of events (S2A and S2B Fig in S1 File, respectively). A non-significant trend of CAC scores was found for lung cancer-specific mortality (Gray’s test, p = 0.2652) (S3 Fig in S1 File).

**Table 2:** 12-year all-cause mortality Cox proportional hazards regression models stratified by automated AI-based CAC score.

	Total	12-year all-cause deaths	Univariate Model HR (95%CI)	Multivariate Model^a^ HR (95%CI)
Automated CAC score	N = 2239	N = 191 (8.5%)		
**0**	125	4 (3.2%)	Ref	Ref
**1–10**	698	34 (4.9%)	1.53 (0.54–4.31)	1.58 (0.56–4.48)
**11–100**	730	58 (8.0%)	2.53 (0.92–6.97)	2.31 (0.83–6.42)
**101–400**	393	45 (11.5%)	**3.71 (1.34–10.32)**	2.74 (0.97–7.71)
**>400**	293	50 (17.1%)	**5.75 (2.08–15.92)**	**3.80 (1.35–10.74)**

HR, hazard ratio; CI, Confidence Interval; CAC, Coronary Artery Calcium

^a^Adjusted for age, sex, smoking status, pack-years, body mass index, and prior cardiovascular disease.

**Table 3:** 12-year non-cancer mortality Fine and Gray’s model stratified by automated AI-based CAC score.

	Total	12-year non-cancer deaths	Univariate Model SHR (95%CI)	Multivariate Model^a^ SHR (95%CI)
Automated CAC score	N = 2239	N = 67 (3.0%)		
**0**	125	1 (0.8%)	Ref	Ref
**1–10**	698	7 (1.0%)	1.25 (0.15–10.22)	1.22 (0.15–10.09)
**11–100**	730	21 (2.9%)	3.62 (0.49–27.04))	3.11 (0.42–23.09)
**101–400**	393	14 (3.6%)	4.51 (0.59–34.44)	3.17 (0.42–24.00)
**>400**	293	24 (8.2%)	**10.62 (1.43–78.98)**	6.76 (0.94–48.77)

SHR, sub-distribution hazard ratio; CI, Confidence Interval; CAC, Coronary Artery Calcium

^a^Adjusted for age, sex, smoking status, pack-years, body mass index, and prior cardiovascular disease.

## Discussion

Fully automated AI-based CAC scoring was an independent predictor of 12-year all-cause mortality in the MILD LCS trial, with a 2.3-fold risk for CAC > 100 compared to ≤ 100 (p < 0.001) and a 2.5-fold risk for CAC > 400 compared to ≤ 400 (p < 0.001). Otherwise, automated CAC scoring did not independently predict 12-year non-cancer mortality and was found not to be significantly associated with lung cancer mortality. CAC values were significantly higher in males than in females, and the CAC scoring increased as the age or the pack-years increased, with its predictive value being similar in males and females despite the different frequencies in the two groups.

Increasing CAC scores were significantly associated with a higher risk of coronary events and all-cause mortality in the NELSON trial [11]. A recent meta-analysis including data from six LCS trials found that subjects with CAC > 400 or > 1000 had more than 2-fold increased relative risk of all-cause mortality compared to lower CAC scores [2]. In a previous study performed on a subset of MILD volunteers, manual CAC predicted all-cause mortality even when adjusted for potential confounders [3]. Our results are mostly in keeping with such evidence, notably regarding all-cause mortality risk stratification, while the estimates of non-cancer mortality were possibly affected by the small number of events. Overall, based on the extended follow-up period, the present analysis highlights the potential for systematic CAC scoring implementation in LCS trials to improve preventive strategies by individual mortality risk assessment.

There is evidence in the literature to suggest a link between CAC and cancer mortality [12, 13]. In a retrospective analysis including 55,943 patients from the CAC consortium, CAC scores were significantly associated with an increased risk of long-term mortality from lung cancer, with the strongest associations for current and former smokers, especially in women [14]. Mirbolouk et al. found an almost two times higher cancer mortality risk in smokers with a CAC ≥ 400 compared with smokers free of CAC [15]. By assessing competing long-term risks of CV disease versus cancer mortality over a median of 12.4 years, Whelton et al. found patients with CAC ≥ 300 have a risk of cancer mortality 1.3-fold higher than patients with a CAC of 0 [16]. In the Multi-Ethnic Study of Atherosclerosis (MESA), CAC scores significantly predicted cancer in both sexes, notably with lung and colorectal cancer showing a stronger association when compared with sex-specific cancers [17]. Our preliminary findings on lung cancer mortality seem not to corroborate previous evidence but need to be interpreted with caution due to the single-center study cohort and the setting of LCS. Further analyses are foreseen to explore the association between CAC and cancer mortality in the MILD trial, ideally providing information on possible links that go beyond lung cancer by including other types of neoplasms diagnosed in the present cohort.

We observed that ex-smokers had significantly higher CAC scores than current smokers. Previous studies reported that smoking cessation is associated with a lower prevalence of CAC compared with current smokers [18], suggesting that it might play a role in decreasing the CAC burden, in apparent discrepancy with our findings. However, the MILD population included ex-smokers who had stopped smoking less than 10 years before to the observation, enabling speculation that a longer duration of smoking cessation may have led to a CAC burden decrease in former smokers [19].

We build on previous methodologies through fully automated AI-based software, possibly increasing reproducibility while reducing the time-consuming manual evaluation process [20, 21]. AI-based approaches have been increasingly employed in CAC evaluation as supported by data demonstrating automated CAC reliable performances compared with the reference standard of manual assessment [4, 22–24]. In the present study, we employed an AI-based software validated on non-electrocardiogram (ECG)–gated LDCT using multi-institutional datasets with manual CAC scoring as the reference standard [25]. The same software yielded better diagnostic performance with 1-mm than 2.5-mm LDCT images [9], supporting our method of analyzing the thinnest reconstruction available. It is worth emphasizing that this approach may lead to potential prognostic implications for participants in LCS trials, that is, in a setting where neither recommended image reconstruction [26] nor ECG-gating is routinely available.

The present study has some limitations. First, the single-center retrospective design is prone to confounding factors affecting the results’ generalizability, such as patient selection. Furthermore, as the MILD study primarily targeted LC, conventional CV risk factors were mostly unknown, thereby not allowing an exhaustive cardiovascular risk profiling. However, measuring risk factors such as lipid levels is not part of current LCS protocols and would affect the logistics of screening practice. Last, data about previous therapies were not systematically collected, preventing a thorough analysis and adjustment of automated CAC prediction.

In conclusion, a fully automated CAC by means of AI-based commercially available software could be performed on chest LDCT for mortality risk stratification in LCS.

## Supporting information

S1 File (DOCX)

S1 Fig (TIF)
