# Supplementary material for: Fully automated calcium scoring predicts all-cause mortality at 12 years in the MILD lung cancer screening trial
Source: PLoS One. 2023 May 16;18(5):e0285593. doi: 10.1371/journal.pone.0285593 (PMC10187890; doi:10.1371/journal.pone.0285593)
Supplement: S1 File — (DOCX) [file pone.0285593.s001.docx]

**Supplementary Material**

**Figure S1.** 12-year all-cause mortality curves stratified by automated CAC scores in (A) males and in (B) females.

**Figure S2.** 12-year non-cancer mortality curves stratified by automated CAC scores in (A) males and in (B) females.

**Figure S3.** 12-year lung cancer mortality curves stratified by automated CAC scores.

**Table S1.** Hazard ratios with 95% confidence intervals of potential confounders included in the multivariate 12-year all-cause mortality Cox proportional hazards regression model.

|  | **Multivariate Model**  **HR (95%CI)** |
| --- | --- |
| **Age** |  |
| **< 60** | Ref |
| **≥ 60** | **2.99 (2.19-4.08)** |
| **Sex** |  |
| **Female** | Ref |
| **Male** | 1.37 (0.95-1.99) |
| **Smoking Status** |  |
| **Ex Smoker** | Ref |
| **Current Smoker** | **1.40 (1.01-1.93)** |
| **Pack-years** |  |
| **<30** | Ref |
| **≥30** | 1.47 (0.98-2.20) |
| **Prior CVD disease** |  |
| **No** | Ref |
| **Yes** | 1.46 (0.99-2.16) |
| **BMI** | 0.97 (0.93-1.01) |

HR, hazard ratio; CI, Confidence Interval; CAC, Coronary Artery Calcium; CVD, Cardiovascular Disease (i.e., angina, myocardial infarction, stroke, or thrombosis); BMI, Body Mass Index.
