# Supplementary figures and images for: Fully automated calcium scoring predicts all-cause mortality at 12 years in the MILD lung cancer screening trial
Source: PLoS One. 2023 May 16;18(5):e0285593. doi: 10.1371/journal.pone.0285593 (PMC10187890; doi:10.1371/journal.pone.0285593)

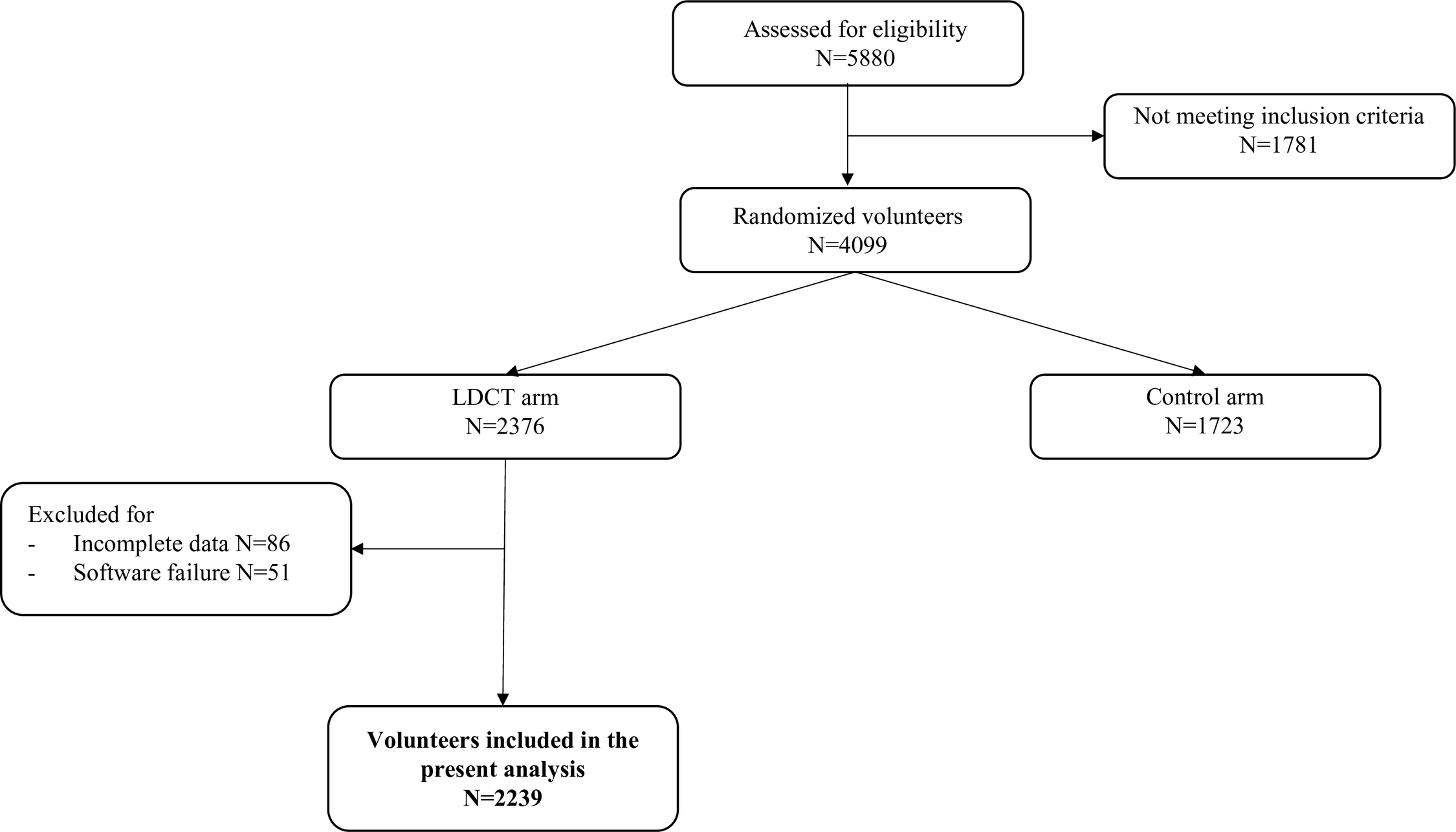

Supplement: S1 Fig — (TIF) [file pone.0285593.s002.tif]
